# Molecular Typing of Methicillin Resistant *Staphylococcus aureus* Clinical Isolates on the Basis of Protein A and Coagulase Gene Polymorphisms

**DOI:** 10.1155/2014/650328

**Published:** 2014-05-15

**Authors:** Nancy Younis Omar, Hala Abdel Salam Ali, Reem Abdel Hameed Harfoush, Engy Hamdy El Khayat

**Affiliations:** Medical Microbiology & Immunology Department, Faculty of Medicine, Alexandria University, El Khartoom Square, El Azarita, Alexandria 21521, Egypt

## Abstract

Increased frequency of methicillin-resistant *Staphylococcus aureus* (MRSA) in hospitalized patients requires rapid and reliable characterization of isolates for control of MRSA spread in hospitals. This study evaluated polymerase chain reaction-restriction fragment length polymorphism (PCR-RFLP) as a molecular typing technique for MRSA strains on the basis of protein A (*spa*) and coagulase (*coa*) gene polymorphisms to verify their ability in assessing the relatedness of isolates. Seventy-five MRSA isolates, from different ICUs of Alexandria University Main Hospital, were characterized using antibiotyping and PCR-RFLP analysis of *coa* and *spa* genes. Thirty-two antibiotypes were identified. *coa* gene PCR generated 3 types and 10 subtypes of band patterns. *HaeIII* restriction digestion of amplified *coa* gene products produced 5 major banding patterns and 12 subtypes. *spa* gene PCR products generated 4 major and 11 minor types, and their *HaeII* restriction digestion showed 5 major and 12 minor banding patterns. The combined *coa* and *spa* RFLP patterns generated 22 combined R types. Typing using *coa* PCR and PCR-RFLP had the same discriminatory index (DI) value (0.64), which was comparable to that of both *spa* PCR and PCR-RFLP techniques (0.68). The combined grouping increased the DI value to 0.836. The current study revealed that testing for multiple gene polymorphisms is more useful for local epidemiologic purposes.

## 1. Introduction

The health risks associated with MRSA infections warrant the implementation of monitoring programs to control its dissemination, given the potential of MRSA to produce invasive infections, particularly in vulnerable patients, and its multiple resistances to antibiotics, which limits the therapeutic options available [1].

Antibiotyping and molecular typing are key functions for epidemiological investigation of hospital-onset* S. aureus *infection [2].

The antibiogram has been the main typing tool in many hospital outbreaks since the technique is widely available and standardized, and it can be used with all microbial species. Its main disadvantage consists of the variability in resistance expression, which is also susceptible to instability due to horizontal transmission and loss of extrachromosomal genetic elements [1].

Molecular methods for MRSA typing as pulsed field gel electrophoresis (PFGE) have good discriminatory power and more reproducibility but are expensive, not widely available, and time consuming [3]. A method for molecular typing of MRSA that depends on polymerase chain reaction-restriction fragment length polymorphism (PCR-RFLP) has proven absolute typeability, reproducibility, and good discriminatory power [4].

Variations in the sequence of genes coding for two species-specific proteins, coagulase (*coa*) and staphylococcal protein A (*spa*), have been the basis for the most widely used forms of PCR typing for* S. aureus*, showing a good correlation with PFGE typing [1].

The coagulase gene amplification discriminatory power relies on the heterogeneity of the region containing the 81 bp tandem repeats at the 3′ coding region of the coagulase gene which differs both in the number of tandem repeats and the location of* AluI* and* HaeIII* restriction sites among different isolates [5]. Protein A has been coded by* spa *gene, where the repeated part is located at 3′end and identified as X region; the repetitive part of region X consists of up to 12 units each with a length of 24 nucleotides. This 24-nucleotide region is highly polymorphic with respect to the number and sequence of repeats. Diversity of X region causes protein A variation [6]. In addition to its use as a marker, the number of repeats in the region X of* spa *has been related to the dissemination potential of MRSA, with higher numbers of repeats associated with higher epidemic capability [1].

The aim of this study was to evaluate PCR-RFLP as a molecular typing technique for MRSA strains on the basis of protein A and coagulase gene polymorphisms and to verify their ability in assessing the relatedness of MRSA isolates.

## 2. Material and Methods

The material of the study consisted of a total of 100* S. aureus* strains isolated from various clinical specimens obtained from patients attending different ICUs in Alexandria Main University Hospital (AMUH) and delivered to the routine laboratory of Microbiology Department at AMUH. All strains were identified as* S. aureus *according to the standard microbiological techniques [7].

### 2.1. Antimicrobial Susceptibility Testing and Detection of MRSA

Fully identified* S. aureus* strains were subjected to antimicrobial susceptibility testing by the Modified Kirby Bauer Disc diffusion method according to the CLSI guidelines (2011) [8], using the following discs: Penicillin (10 U), oxacillin (1 *μ*g), cefoxitin (30 *μ*g), erythromycin (15 *μ*g), gentamicin (10 *μ*g), tetracycline (30 *μ*g), ciprofloxacin (5 *μ*g), vancomycin (30 *μ*g), trimethoprim/sulfamethoxazole (25 *μ*g), amikacin (30 *μ*g), clindamycin (2 *μ*g), chloramphenicol (30 *μ*g), rifampicin (5 *μ*g), and teicoplanin (30 *μ*g) (Oxoid). MRSA strains were further defined as multidrug-resistant (MDR), extensively drug-resistant (XDR), or pandrug-resistant (PDR), guided by the categorization system proposed by the European Society of Clinical Microbiology and Infectious Diseases in 2011 [9]. A standard MSSA strain (ATCC 25923) and a MRSA strain (ATCC 33591) were used as control.

### 2.2. PCR to Detect mecA Gene [10]

All* S. aureus* isolates were tested for the presence of the 310 base pair (bp) PCR product of* mecA* gene, using the following primers: forward (5′-TGG CTA TCG TGT CAC AAT CG-3′) and reverse (5′-CTG GAA CTT GTT GAG CAG AG-3′).* MecA* positive strain (ATCC 33591) was included as positive control. Amplification reaction was carried out in 25 *μ*L volume, under the following conditions: initial denaturation at 92°C for 5 min, followed by 35 cycles of denaturation at 95°C for 30 seconds, annealing at 56°C for 30 seconds, and extension at 72°C for 30 seconds, followed by final extension at 72°C for 3 min.

### 2.3. MRSA Typing

*(A) PCR of coa Gene [5]*. The identified MRSA strains were subjected to PCR for* coa* gene detection, using the following primers: forward (5′-CGA GAC CAA GAT TCA ACA AG-3′) and reverse (5′-AAA GAA AAC CAC TCA CAT CA-3′), which were designed to amplify the 3′ end hyper variable region containing 81 bp tandem repeats of* coa* gene. The amplification reaction consisted of an initial denaturation step at 94°C for 5 min, followed by 30 cycles of denaturation at 95°C for 30 sec, annealing at 55°C for 45 sec, extension at 72°C for 2 min, followed by final extension at 72°C for 7 min.

*RFLP of coa Gene PCR Products [5]*. Depending on the number of 81 bp repeats, a strain analysis of PCR RFLP products was performed with* HaeIII* restriction enzyme (New England BioLabs, Frankfurt, Germany), where 10 *μ*L of PCR product of* coa* gene was incubated with 6 U of the enzyme at 37°C for 1 h 45 min in a water bath.

*(B) PCR of spa Gene [4].* The identified MRSA strains were subjected to PCR for* spa* gene detection using the following primers: forward (5′-ATC TGG TGG CGT AAC ACC TG-3′), and reverse (5′-CGC TGC ACC TAA CGC TAA TG-3′) which were designed to amplify the polymorphic X region that contains a variable number of 24 bp tandem repeats of the* spa* gene coding for protein A. Amplification reaction consisted of an initial denaturation step at 94°C for 4 minutes,followed by 35 cycles of denaturation at 94°C for 1 minute, annealing at 56°C for 1 minute, extension at 72°C for 3 minutes, followed by final extension at 72°C for 5 minutes.

*RFLP of spa Gene PCR Products [4, 5].* Five *μ*L of each* spa* gene amplicon and 10 units of* HaeII *restriction enzyme (New England BioLabs, Frankfurt, Germany) were incubated at 37°C for 3 hours. The PCR products and restriction digest fragments were detected by electrophoresis in 2% agarose gel.

The interpretation criteria for identifying different strains were a single band difference. Unique PCR-RFLP patterns were assigned a genotype.

### 2.4. Statistical Analysis: Determination of Numerical Index of Discrimination

The probability that two unrelated isolates sampled from the test population will be placed into different typing groups or clusters was assessed according to the Hunter-Gaston formula [11]. This probability, also called discriminatory index, is calculated using the following equation:

(1) $$\begin{array}{c} D=1-\frac{1}{N\left(N-1\right)}\overset{S}{\underset{j=1}{\sum }}n_{j}\left(n_{j}-1\right), \end{array}$$

where *D* = numerical index of discrimination, *N* = the total number of isolates in the sample population, *S* = the total number of types obtained, and *n*_*j*_ = the number of isolates belonging to this type.

## 3. Results

**Figure 1:**
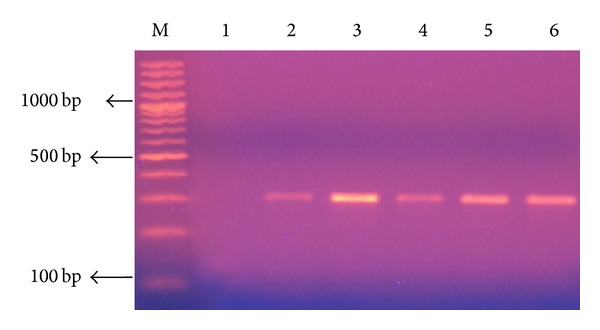
2% Agarose gel electrophoresis analysis of PCR amplification products of* mecA* gene of 310 bp, extracted from* S. aureus*. Lane1: negative control (no DNA template); lane 2: positive control (*mecA* positive strain ATCC 33591); lanes 3–6: methicillin-resistant* S. aureus* (MRSA); lane M: DNA molecular size marker (100 bp ladder).

A total of 100* S. aureus *strains isolated from different clinical specimens and different ICUs were identified according to the standard microbiological techniques. MRSA represented 75% of total* S. aureus* isolates, while MSSA represented 25% of the strains. Differentiation was based on sensitivity testing using oxacillin and cefoxitin discs, confirmed by the detection of the amplified 310 bp* mecA *gene using PCR in 75 MRSA strains (Figure 1).

### 3.1. Distribution of MRSA with Respect to Clinical Specimen Type, Admission ICU, Age, and Sex

Among the 75 MRSA isolates, the highest number of isolates was in sputum and tracheal aspirates (46.7%), followed by pus (29.3%), blood cultures (16%), and then urine (6.7%). A single MRSA isolate was isolated from a nasal swab. The highest percentage of MRSA isolates was from ICU 3 (44%), followed by ICU 1 (30.6%), ICU 7 (22.6%), and ICU 6 (2.6%). The age of patients ranged between 15 years and 70 years. The highest percentage of MRSA isolates fell in the age group from >40 to 50 years (26.7%) followed by (21.3%) in age group >30–40 years and (20%) in >20–30 years. Forty-six (61.3%) of MRSA isolates were recovered from male patients.

### 3.2. Antimicrobial Susceptibility Testing and Antibiotypes of MRSA Isolates

All 75 MRSA isolates were resistant to penicillin, oxacillin, and cefoxitin and all were sensitive to vancomycin and teicoplanin. Resistance to tetracycline, gentamicin, ciprofloxacin, amikacin, erythromycin, chloramphenicol, and clindamycin was high (94.7%, 93.3%, 92%, 89.3%, 73.3%, 69.3%, and 65.3%, resp.), while resistance to rifampin was 36% and that to trimethoprim-sulfamethoxazole was the lowest (18.7%).

Thirty-two antibiotypes were identified. The most common antibiotypes (1 and 2) were found in 16% (12/75) and 10.6% (8/75) of the isolates, respectively. Antibiotype 1 isolates were sensitive to trimethoprim-sulfamethoxazole, intermediately sensitive to rifampin, and resistant to erythromycin, gentamicin, tetracycline, amikacin, chloramphenicol, clindamycin, and ciprofloxacin. Antibiotype 2 isolates were similar to those of antibiotype 1 except that they were sensitive to rifampin. The DI of the antibiogram was (0.94).

### 3.3. MRSA Genotyping

**Figure 2:**
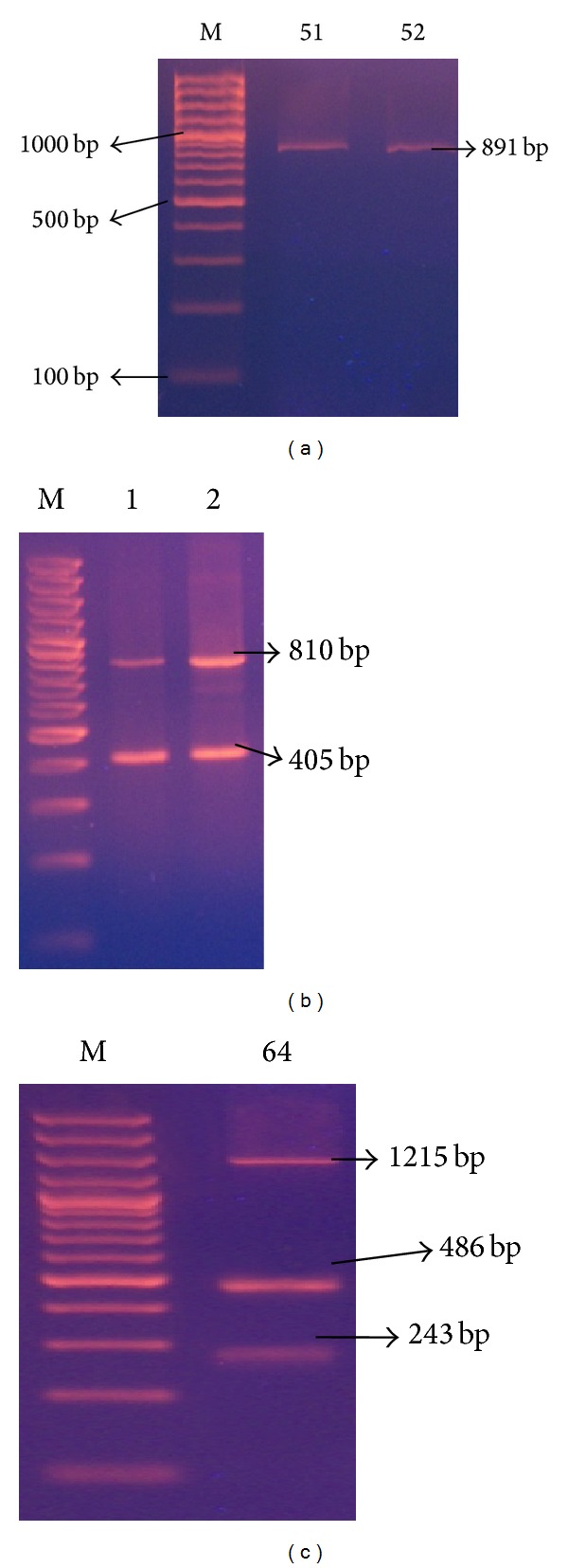
Representative 2% agarose gel electrophoresis of* coa* gene PCR products where M is DNA molecular size marker (100 bp ladder), (a) isolates 51-52 showing single band, (b) isolates 1-2 showing 2 bands, and (c) isolate 64 showing 3 bands* coa *gene PCR products.

*(1) Coagulase Gene Typing.* PCR products of 9 sizes, ranging from 81 to 1215 bp in increments of 81 bp (81, 243, 405, 486, 729, 810, 891, 972, and 1215 bp), were obtained. Pattern electrophoresis analysis generated 3 different types (Co1, Co2, and Co3) and 10 subtypes of band patterns. The majority of MRSA strains (54/75) showed double bands (type Co2), 5/75 showed three bands (type Co3), and the remaining 16/75 strains showed a single PCR band (type Co1) (Table 1 and Figure 2). The most common PCR* coa* gene product shown was the 405 bp band size product. DI value of* coa* gene PCR was 0.64.

**Figure 3:**
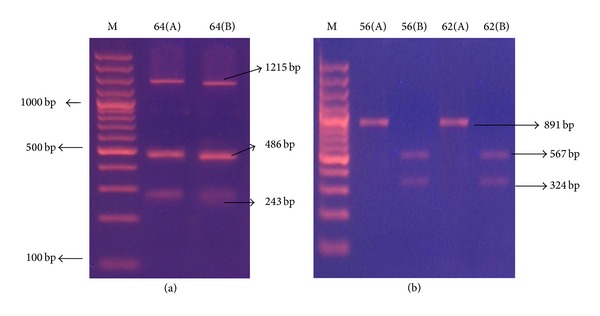
Representative 2% agarose gel electrophoresis of* coa* gene* HaeIII* restriction enzyme digestion PCR products, where M is DNA molecular size marker (100 bp ladder), (a) isolate 64 showing 3 bands* coa* gene PCR products (A) that remained uncut and after (B) cutting with* HaeIII* restriction enzyme, (b) isolates 56 and 62 showing single band* coa* gene PCR product (A) and their corresponding 2 bands* HaeIII* restriction digestion (B).

*(2) coa-RFLP Typing Using HaeIII Restriction Enzyme.* Restriction digestion was performed on the amplified coagulase PCR products with* HaeIII*. The bands produced were multiples of 81, divided into 9 band classes. Five distinct RFLP banding patterns (designated as A, B, C, D, and E) and 12 subtypes designated as [A1,2,3/B/C1, 2, 3/D1, 2, 3/E1, 2] were obtained (Table 1 and Figure 3). The majority of strains (44/75 = 58.6%) belonged to RFLP banding pattern D1. PCR products of 4 strains were not digested by* HaeIII*. Accordingly, this method had 94.6% typeability. DI value of* coa* PCR-RFLP was 0.64.

**Figure 4:**
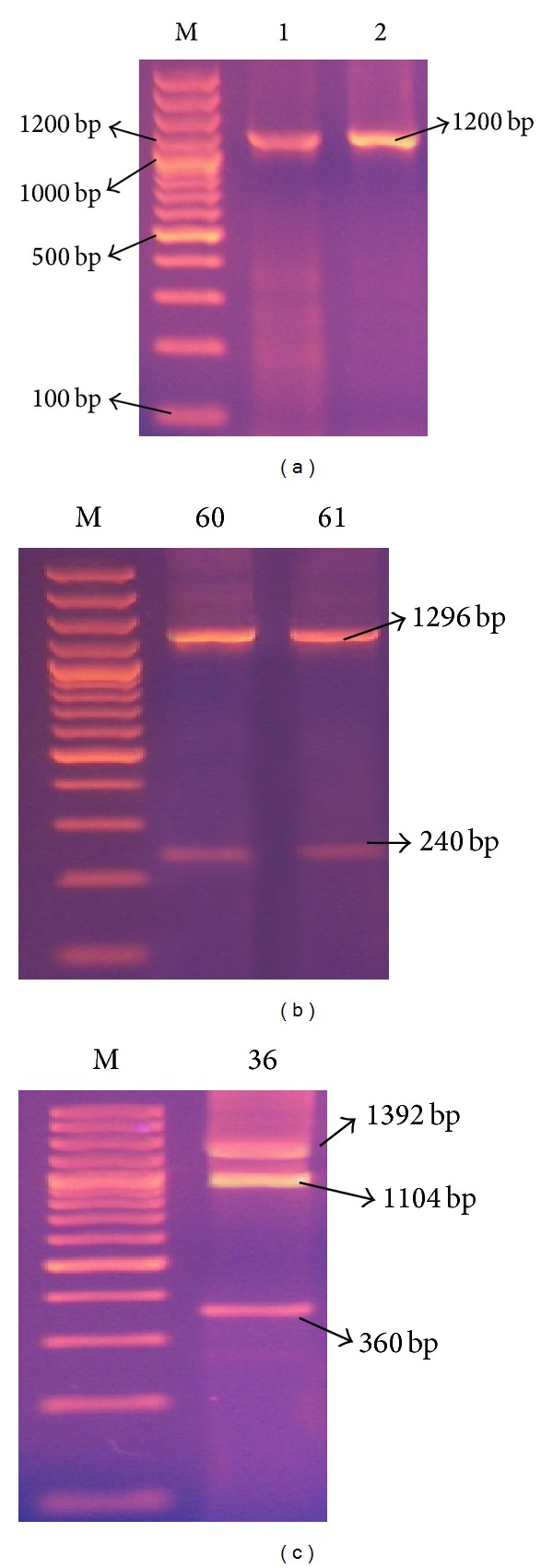
Representative 2% agarose gel electrophoresis of* spa* gene PCR products where M is DNA molecular size marker (100 bp ladder). (a) Isolates 1 and 2 showing single band PCR products, (b) isolates 60 and 61 showing 2 bands PCR products, and (c) isolate 36 showing 3 bands PCR products.

*(3) spa Gene Typing*.* spa* gene PCR products of different sizes could be detected in 71 out of the 75 MRSA isolates. The size of the PCR products ranged from 144 to 1392 bp (144, 192, 240, 360, 408, 600, 1104, 1200, 1296, and 1392), reflecting the number of 24 bp repeat units contained in the* spa *gene. These PCR products generated 4 major and 11 minor different* spa* gene types. Sixty-three MRSA strains (84%) showed single PCR band (type S1), 7 (9.3%) had two bands (type S2), and only one (1.3%) strain showed 3 PCR bands (type S3) (Table 2 and Figure 4). The absence of PCR product is considered a separate type and this was shown in 4 strains (S4). DI value of* spa* gene PCR typing was 0.68.

**Figure 5:**
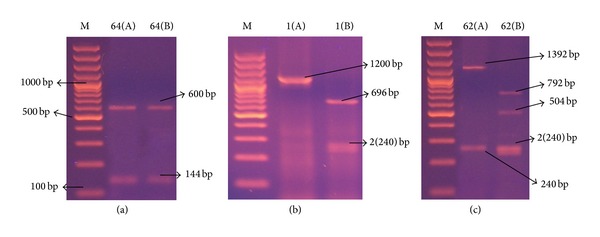
Representative 2% agarose gel electrophoresis of* spa* gene* HaeII* restriction enzyme digestion PCR products, where M is DNA molecular size marker (100 bp ladder). (a) isolate 64 showing 2 bands* spa* gene PCR products (A) that remained uncut after (B) restriction, (b) isolate 1 showing single band* spa* gene PCR products (A) and the corresponding 3 bands* HaeII* restriction digestion pattern (B), and (c) isolate 62 showing 2 bands* spa* gene PCR products (A) and its corresponding 4 bands* HaeII* restriction digestion products (B).

*(4) spa-RFLP Typing and HaeII Restriction Enzyme Digestion.* Restriction digestion performed on the amplified* spa* PCR products with* HaeII* revealed bands observed to be multiples of 24. Five distinct banding patterns (designated as A, B, C, D, and E) and 12 subtypes designated as [A1, 2/B1, 2, 3, 4, 5/C1, 2, 3/D/E] were produced (Table 2 and Figure 5). Most strains (65/75 = 86.6%) belonged to pattern B especially subtypes B4 (49.3%) and B2 (28%). PCR products of 2 strains were not digested by* HaeII* and 4 strains had no* spa* gene band; therefore, this method had 92% typeability. DI value of* spa* gene RFLP typing method was 0.68.

*(5) coa and spa RFLP Combined Analysis (Table 3).* The combination of both RFLP patterns was able to identify 22 types (R1–R22), where types R2 and R1 were the most frequent, including 27 and 13 MRSA isolates, respectively. Thirty-three (44%) MRSA strains were from ICU 3, 23 (30.6%) MRSA strains were from ICU 1, 17 (22.6%) from ICU 7, and only 2 (2.6%) from ICU 6. Of the 33 samples from ICU 3, 45.5% of MRSA strains (15/33) were of type R2 and 6/33 (18%) strains belonged to type R1. Concerning the 23 isolates of ICU 1, 34.7% isolates (8/23) were of type R2 and 21.7% (5/23) were of type R1. The remaining isolates were distributed among the different types. The DI value of* HaeII* and* HaeIII* combined RFLP classification was 0.836.

DI value of* coa *PCR-RFLP was not different from DI value of* coa* gene PCR product (0.64). DI value of* spa* gene PCR typing was the same as that of* spa* PCR-RFLP (0.68). DI value of the antibiotyping was the highest (0.948). DI value of* HaeII* and* HaeIII* combined classification was 0.836 which is higher than the highest of individual typing (Table 4). So it is desirable that RFLP of both genes should be used for better and reliable discrimination.

## 4. Discussion

MRSA is the major cause of nosocomial mortality and morbidity; it is commonly found in the community and hospital environment especially in the ICUs [12].

The present study was conducted on patients attending different ICU wards in AMUH. The backgrounds of the patients (ICU, age, and sex) were randomized to reduce the possibility of contact transmission and various specimens were used for MRSA isolation (respiratory system fluid or secretions, pus, blood, urine, and nasal swabs). Among 100* S. aureus* isolated strains, 75 were proven to be MRSA by disc diffusion detection of* mecA* gene by PCR. These isolates were selected for further genotypic characterization.

Among the 75 MRSA strains, 35 (46.7%) were isolated from respiratory system, 22 (29.3%) from wounds, 12 (16%) from blood samples, 5 (6.7%) from urine samples, and only one sample (1.3%) from a colonized nose. Schmitz et al. [13], in Germany, found that the greatest proportion (17.6%) of their MRSA isolates was from the bronchial/tracheal aspirates.

None of our isolates was resistant to vancomycin or teicoplanin, but all were resistant to penicillin, with variable resistance patterns to the used antimicrobial panel. Using a different panel with few similar antibiotics, Baddour et al. [14] study showed different susceptibility patterns. The difference noted in the susceptibility of the MRSA isolates from different hospitals probably reflects the different patterns of antibiotic usage and thus development of resistance in these hospitals. Antibiotic resistance patterns are influenced by the local environment, selective antibiotic pressure, acquisition, and loss of plasmids carrying resistance genes and various other genetic mechanisms [15]. Sixteen of our isolates could be classified as extensively drug-resistant (XDR). The remaining 59 are multidrug-resistant (MDR) and none was pandrug-resistant (PDR). Other studies documented the association of recovery of MDR-MRSA strains from inpatient clinical samples rather than from outpatients [16, 17].

MRSA typing is an essential component of an effective surveillance system to describe epidemiological trends and infection control strategies. Antibiogram typing has been successfully used for screening of epidemic strains [15]. In the present study, 32 antibiotypes were identified.

Janwithayanuchit et al. [18] from Thailand identified 9 different antibiotypes using a panel of 10 antimicrobial agents. Antibiotypes 1 and 2 represented 44.2% (57/129) and 35.6% (46/129) of their isolates, respectively. Those were nearly similar to our antibiotypes 1 and 2 except in sensitivity to trimethoprim-sulfamethoxazole. They concluded that antimicrobial susceptibility testing is the simplest epidemiologic typing method, the results are easy to interpret, and minimal laboratory skills and equipment are required. Nevertheless, its main disadvantage consists of the variability in resistance expression, which is susceptible to instability due to loss of extra chromosomal genetic elements. However, antibiogram typing cannot be used as the only typing method for MRSA because of its poor discriminatory power [1].

Genotypic techniques to type MRSA must be particularly discriminatory, as MRSA strains probably originate from a single clone or at least a few strain types [13].

The* coa* gene is the principal criterion for the identification of* S. aureus* isolates [1]. Its 3′ end contains an 81 bp tandem short sequence repeat (SSR) series, the number of which differs between strains [19]. The* coa* gene was identified in all of MRSA isolates in this study, indicating 100% typeability. The* coa* PCR showed 100% sensitivity and specificity in Tiwari et al. (2008) [20] study, confirming the fact that this gene is present in all* S. aureus* isolates.

In the present study, 9 different amplicons of the* coa* gene with band sizes ranging from 81 to 1215 bp were found, generating 3 different types and 10 subtypes of* coa *band patterns. The majority (54/75) of MRSA strains showed double bands, 16 (21.3%) showed single band, and the remaining 5 (6.7%) had three bands. The 405 bp band was the most common and was found in 57/75 of the isolates (76%). The presence of more than one band has been explained by the existence of more than one allelic form of coagulase gene, allowing one strain to produce one or more of these variants [21]. This gene polymorphism might be due to deletion or insertion mutations by which a portion of the 3′ end region of the* coa *gene is deleted or several nucleotides are inserted and as a consequence change the* coa *gene size [22].

Himabindu et al. [5], using the same primer, showed that the sizes of* coa* PCR products were classified into 3 band classes. The majority of isolates belonged to the band class of 812 bp, which was close to our study results, where 69.3% of our isolates belonged to the same band class (810 bp). The difference in coagulase types was found to be subject to geographical variation [23].

According to Lawrence et al. [24], the high discriminative power of* coa* gene typing in their study was associated with the recovery of a 402 bp PCR product, which was always connected with detection of MRSA in culture. This was in agreement with our finding, where the majority (57 = 76%) of our isolates produced a 405 bp PCR band using the same primers.

In the present study,* HaeIII* restriction enzyme was chosen for* coa* gene PCR-RFLP, aiming at a more detailed characterization of the MRSA isolates. It generated 5 RFLP banding patterns (A, B, C, D, and E) and 12 subtypes [A1, 2, 3/B/C1, 2, 3/D1, 2, 3/E1, 2]. Four strains were not digested by the enzyme, indicating that not all the isolates genomic DNA contained* HaeIII* restriction sites; accordingly, this method had 94.6% typeability.

Lawrence et al. study [24] recovered a 402 bp PCR product which was cut into three fragments of 176 bp, 146 bp, and 81 bp after* HaeIII* digestion. This was different from our findings where the digestion of the PCR product of band size 405 bp by* HaeIII* enzyme was unsuccessful, along with the production of other bands (1215 bp, 891, 486, 243, 81) that also remained uncut which might explain the fewer number of genotypes obtained by* coa *PCR-RFLP in our study.

There is a high degree of heterogeneity in the* coa *gene of* S. aureus* making PCR-RFLP of* coa* gene not suitable as a single typing method [18]. The polymorphisms of the* spa *and* coa* genes were not independent, and combination use of them was found by many researchers to be a valid instrument for epidemiological studies [25].

The* spa *gene is not consistently expressed in all* S. aureus* isolates [26]. In the present study,* spa* gene was expressed in 71 isolates (94.6%). X region of* spa *gene is polymorphic, containing a varying number of 24 bp repeats, whose number and sequence differ among strains [26].

The sizes of the* spa* PCR products in the current study were classified into 11 classes, creating 4 major and 11 minor different* spa* gene types according to band number: 63 strains showed single PCR band, 7 had two bands, and only one strain showed 3 PCR bands. The absence of PCR product is considered a separate type and this was shown in 4 strains. This was also shown in Shakeri et al. [6] and Adesida et al. [27] studies, where* spa* gene was absent in 3.8% and 5% of their* S. aureus* isolates, respectively.

Similar results were obtained by Schmitz et al. [13] and Montesinos et al. [1], who distinguished, by* spa *gene PCR, 5 types amongst the 183 and 4 types amongst 124 of their MRSA isolates, respectively.

In addition to its use as a marker, the number of repeats in the region X of* spa *has been related to the dissemination potential of MRSA, with higher numbers of repeats associated with higher epidemic capability [26].* S. aureus *strains with shorter length of protein A cannot adhere to the surface of nasal epithelium and are discharged by breath, sneezing, and coughing [6]. This observation might explain our findings that MRSA strains with longer* spa* bands (>1200 bp = 1200–1392 bp) were isolated from respiratory secretions (85.6%) more than other samples, reflecting the role of* spa* protein in adherence to the respiratory epithelium, which protects the isolate from being discharged by breath, sneezing, and coughing.

There is also controversy over the proposed correlation between the number of repeats and the MRSA dissemination potential, with most of the epidemic strains reported as having more than seven repeats. The spread of MRSA strains between hospitals has been recognized in several studies, perhaps as a consequence of the prolonged carrier status and the increased mobility of the population [1]. In the present study, all but one spa gene positive MRSA isolates had more than 7 repeats (>168 bp), and all isolates were disseminated in different ICU wards in the hospital.

*HaeII* was the restriction enzyme used to digest* spa* PCR products in the present study, showing 5 distinct* spa *banding RFLP patterns (A, B, C, and D & E) and 12 minor patterns. The middle sized band (240 bp) remained the same in 67/75 isolates (89.3%).

Closer results were reported by Mehndiratta et al. [28], who reported 5 types of spa gene PCR-RFLP patterns of 1150–1420 bp fragments. However, their smallest fragment that remained the same in all patterns was of size 243 bp.

In Wichelhaus et al. study [4], the discriminatory power of PCR-RFLP of* coa* and* spa* genes using* HaeII* enzyme was found to be sufficiently high and corresponded reasonably well with PFGE. Wilailuckana et al. [29] also reported that typing results of MRSA isolates using combinations of different molecular methods provided a considerably usable tool for discrimination.

In the present study, the combination of both* coa *and* spa* genes PCR-RFLP patterns was able to identify 22 combined R types (R1–R22), where types R2 and R1 were the most frequent, including 27 and 13 MRSA isolates, respectively.

A similarly designed study by Mitani et al. [30] reported 8* spa* and 6* coa* types using* HaeII* restriction enzyme for* coa* and* spa *genes PCR-RFLP. Combination of these 2 techniques revealed 10 R types, where types R1–R4 were relatively frequent.

In the present study, typing using both* coa* PCR and* coa *PCR-RFLP had the same DI value (0.64), which is comparable to DI value of typing using both* spa* PCR and* spa* PCR-RFLP (0.68). Moreover, the combined grouping of* coa* PCR-RFLP and* spa* PCR-RFLP increased the discriminatory index of the typing procedure to 0.836 which is higher when compared to the highest DI of the individual typing technique. So it is desirable that RFLP of both genes should be used for better and reliable discrimination.

The DI value of the antibiotyping in the current study was the highest among all techniques used (0.94). The antibiotic abuse and the lack of antibiotic stewardship in our hospitals could explain the lack of correlation between antibiotypes and genotypes of our MRSA isolates.

This study demonstrates that, although PFGE remains the gold standard for MRSA typing, the typeability of PCR-RFLP products in the genotyping of MRSA strains was an attractive tool for routine epidemiological surveillance and infection control. We therefore recommend this method as a reliable and rapid system in epidemiological investigations similar to this study. Our study revealed that no single gene analysis is efficient in distinguishing strains within a heterogeneous species and testing for multiple gene polymorphisms is more useful for local epidemiologic or outbreak investigation purposes. The polymorphisms of the* spa* and* coa* genes are not independently discriminative, and the combined use of both was found to be a valid instrument for epidemiological studies. The variability in genes that are built from repetitive moieties might go along with enhanced speed of evolution. This typing system, therefore, is intended mainly for routine epidemiological surveillance, where isolates obtained within a narrow time frame are subject for typing analysis.

## Figures and Tables

**Table 1:** Combined *coa* gene typing and *HaeIII* RFLP patterns of the 75 isolated MRSA strains.

*coa* band types (3)	*coa* gene subtypes (10)	RFLP pattern (12)	Size of PCR product (approximate bp)	Size of *HaeIII* fragments (approximate bp)	Total isolates number (%)	Isolate serial number
**Co1 (1 band)** 16/75 (21.3%)	**Co1a**	**C1 (3 bands)**	810	2 (162), 486	3 (4)	7, 12, 13
	**Co1b**	**A1 (uncut)**	891	891	2 (2.7)	43, 49
		**B (2 bands)**	891	324, 567	3 (4)	56, 62, 68
		**C2 (3 bands)**	891	2 (162), 567	7 (9)	51–55, 57, 58
	**Co1c**	**A2 (uncut)**	1215	1215	1 (1.3)	46

**Co2 (2 bands)** 54/75 (72%)	**Co2a**	**D1 (4 bands)**	405, 810	2 (162), 405, 486	44 (58.7)	1–6, 9–11, 16, 18–20, 22–28, 30–35, 37–42, 45, 47, 50, 60, 61, 65, 66, 71–75
	**Co2b**	**D2 (4 bands)**	405, 729	81, 162, 405, 486	1 (1.3)	17
	**Co2c**	**D3 (4 bands)**	405, 891	2 (162), 405, 567	7 (9)	44, 48, 59, 63, 67, 69, 70
	**Co2d**	**C3 (3 bands)**	81, 810	81, 2 (405)	1 (1.3)	14
	**Co2e**	**E1 (5 bands)**	405, 972	81, 162, 324, 405, 486	1 (1.3)	36

**Co3 (3 bands)** 5/75 (6.7%)	**Co3a**	**E2 (5 bands)**	81, 405, 810	81, 2 (162), 405, 486	4 (5.3)	8, 15, 21, 29
	**Co3b**	**A3 (uncut)**	243, 486, 1215	243, 486, 1215	1 (1.3)	64

**Table 2:** Combined *spa* gene typing and *HaeII* RFLP patterns of the 75 isolated MRSA strains.

*spa* band types (4)	*spa* gene subtypes (11)	RFLP pattern By *HaeII* (12)	Size of PCR product (approximate bp)	Size of *HaeII* fragments (approximate bp)	Total isolates number (%)	Isolate serial number
**S1 (1 band)** 63/75 (84%)	**S1a**	**A1 (uncut)**	192	192	1 (1.3)	43
	**S1b**	**B1**	1104	2 (240), 600	3 (4)	44, 69, 70
	**S1c**	**B2**	1200	2 (240), 696	21 (28)	1–3, 15, 17, 45, 47, 48, 50, 57–59, 63, 66, 67, 71–75
		**B3**	1200	2 (192), 792	1 (1.3)	55
	**S1d**	**B4**	1392	2 (240), 912	37 (49.3)	4–13, 18–35, 37, 38, 40–42, 51–54

**S2 (2 bands)** 7/75 (9.3%)	**S2a**	**A2 (uncut)**	144, 600	144, 600	1 (1.3)	64
	**S2b**	**C1**	408, 1296	2 (192), 312, 1008	1 (1.3)	14
	**S2c**	**B5**	240, 1200	2 (240), 912	3 (4)	60, 61, 65
	**S2d**	**C2**	240, 1392	2 (240), 504, 792	1 (1.3)	62
	**S2e**	**C3**	360, 1392	100, 240, 504, 912	1 (1.3)	68

**S3 (3 bands)** 1/75 (1.3%)	**S3**	**D**	360, 1104, 1392	150, 2 (240), 792, 456, 912	1 (1.3)	36

**S4 (no band)** 4/75 (5.3%)	**S4** **No band**	**E**	—	—	4 (5.3)	39, 46, 49, 56

**Table 3:** Analysis of the discriminatory power of the binary typing method (R) compared with the *coa* and *spa* genotyping techniques and antibiogram estimated on the basis of the typing results for the 75 MRSA strains.

R Type (*n*)	*coa *type/RFLP pattern	*spa *type/RFLP pattern	Isolate origin	Antibiotype
**R1 (13)** (5) = ICU 1 (6) = ICU 3 (2) = ICU 7	Co2a/D1	S1c/B2	Sputum (3) Pus (6) Blood (2) Pus (6) Mini-BAL (1) Urine (1)	1–3, 7, 19, 20, 30–32

**R2 (27)** (8) = ICU 1 (15) = ICU 3 (3) = ICU 7 (1) = ICU 6	Co2a/D1	S1d/B4	Sputum (4) Pus (7) Mini-BAL (9) Blood (3) BAL (2) Urine (2)	1–7, 9, 11–17, 31

**R3 (3)** (1) = ICU 1 (1) = ICU 3 (1) = ICU 7	Co1a/C1	S1d/B4	Wound Pus Mini-BAL	1, 4

**R4 (3)** (1) = ICU 1 (1) = ICU 3 (1) = ICU 7	Co3a/E2	S1d/B4	BAL (2) Sputum (1)	6, 7, 11

**R5**	Co2d/C4	S2b/C1	Bed sore/ICU 7	8

**R6**	Co3a/E2	S1c/B2	BAL/ICU 3	9

**R7**	Co2b/D2	S1c/B2	Nasal swab/ICU 3	10

**R8**	Co2e/E1	S3/D	BAL/ICU 7	2

**R9**	Co2a/D1	S4/E	BAL/ICU 6	15

**R10**	Co1b/A1	S1a/A1	BAL/ICU 1	18

**R11 (3)** (1) = ICU 1 (2) = ICU 3	Co2c/D3	S1b/B1	Sputum (2)	17, 19

**R12**	Co1c/A2	S4/E	Blood/ICU 1	21

**R13 (4)** (2) = ICU 3 (2) = ICU 7	Co2c/D3	S1c/B2	Mini-BAL Urine BAL Blood	1, 2, 19, 27

**R14**	Co1b/A1	S4/E	Wound/ICU 7	22

**R15 (4)** (2) = ICU 1 (1) = ICU 3 (1) = ICU 7	Co1b/C2	S1d/B4	Blood Urine BAL Sputum	1, 2, 6, 23

**R16**	Co1b/C2	S1c/B3	Pus/ICU 7	1

**R17**	Co1b/B	S4/E	Pus/ICU 3	1

**R18 (2)** (1) = ICU 1 (1) = ICU 7	Co1b/C2	S1c/B2	Wound Mini-BAL	1, 24

**R19 (3)** (1) = ICU 1 (1) = ICU 3 (1) = ICU 7	Co2a/D1	S2c/B5	Wound (2) Blood (1)	3, 7, 25 3

**R20**	Co1b/B	S2d/C2	Blood/ICU 3	26

**R21**	Co3b/A3	S2a/A2	Blood/ICU 1	28

**R22**	Co1b/B	S2e/C3	Blood/ICU 7	29

**Table 4:** Discriminatory indices (DIs) of the binary typing method (R) compared with the *coa* and *spa* genotyping techniques and antibiogram for the 75 MRSA strains.

Method	Types	Size (%) of largest type	Discriminatory index (DI)
*coa* gene PCR typing	10	44 (58.7%)	0.64
*spa* gene PCR	11	37 (49.3%)	0.68
*coa*/*HaeIII* RFLP	12	44 (58.7%)	0.64
*spa*/*HaeII* RFLP	12	37 (49.3%)	0.68
Binary typing (R)	22	27 (36%)	0.836
Antibiotyping	32	12 (16%)	0.948
